# COVID-19—The Shift of Homeostasis into Oncopathology or Chronic Fibrosis in Terms of Female Reproductive System Involvement

**DOI:** 10.3390/ijms24108579

**Published:** 2023-05-11

**Authors:** Elena Petersen, Daria Chudakova, Daiana Erdyneeva, Dulamsuren Zorigt, Evgeniya Shabalina, Denis Gudkov, Pavel Karalkin, Igor Reshetov, Ospan A. Mynbaev

**Affiliations:** 1 Moscow Institute of Physics and Technology, 141701 Dolgoprudny, Russia; 2 P.A. Herzen Moscow Research Institute of Oncology, 125284 Moscow, Russia; 3 Institute of Cluster Oncology, I.M. Sechenov First Moscow State Medical University, 119991 Moscow, Russia

**Keywords:** COVID-19, SARS-CoV-2, tumor microenvironment, tissue microenvironment, malignant tumors, oncogenic viruses, female reproductive system, endometrial cancer, uterine fibrosis

## Abstract

The COVID-19 pandemic caused by the SARS-CoV-2 coronavirus remains a global public health concern due to the systemic nature of the infection and its long-term consequences, many of which remain to be elucidated. SARS-CoV-2 targets endothelial cells and blood vessels, altering the tissue microenvironment, its secretion, immune-cell subpopulations, the extracellular matrix, and the molecular composition and mechanical properties. The female reproductive system has high regenerative potential, but can accumulate damage, including due to SARS-CoV-2. COVID-19 is profibrotic and can change the tissue microenvironment toward an oncogenic niche. This makes COVID-19 and its consequences one of the potential regulators of a homeostasis shift toward oncopathology and fibrosis in the tissues of the female reproductive system. We are looking at SARS-CoV-2-induced changes at all levels in the female reproductive system.

## 1. Introduction

SARS-CoV-2, the causative agent of coronavirus disease 2019 (COVID-19), has caused widespread morbidity and mortality since its emergence in late 2019. The COVID-19 pandemic has become widespread and known as a pathology of the respiratory system, affecting the ciliary epithelium at an early stage [1]. In severe cases, COVID-19 can lead to development of lung disease: acute respiratory distress syndrome (ARDS). A variety of extrapulmonary symptoms may also occur, including acute renal failure (AKI); acute heart failure; coagulopathy; thromboembolic complications, including stroke and pulmonary embolism; and circulatory shock [2,3]. At the same time, data on the impact of COVID-19 on the female reproductive system (FRS) are very scant [4], and they are often fragmented. The similarity of the tissues of the alveolar and endometrial epitheliums, the presence of a large number of capillaries, and an increased level of expression of the ACE2 receptor make it possible to extrapolate the processes described from the lung tissue to the endometrial tissue. At the same time, the FRS is a complex system, the work of which is associated with hormones, the functional state of the endothelium, and the state of the microvascular bed, so not all manifestations of COVID-19 at the tissue level can be equivalent. The FRS is in a delicate balance of all factors that have a regulatory effect on the normal function of the system. Chronic inflammation, epithelial–mesenchymal transition (EMT), and fibrosis play leading roles in the pathogenesis of many conditions of the FRS. COVID-19 acts on these same targets and may shift FRS homeostasis toward a more pathological state. What exactly will be the final outcome—inflammation, fibrotic changes, or precancerous or cancerous pathologies—will be determined by the sum of the factors that ensure homeostasis, as well as its shifts under the influence of SARS-CoV-2. Importantly, in a “post-COVID-19 reality,” a significant number of COVID-19 survivors, estimated as about 40% of survivors, are expected to be affected by COVID-19 sequelae or a post-COVID-19 syndrome (PCS, also known as Long COVID-19)—systemic, multiorgan-targeting consequences of COVID-19, changing affected tissues, potentially triggering dormant diseases, and creating a tissue microenvironment that promotes other pathologies [5]. It is already known that COVID-19 impacts the FRS [4], although the long-term consequences of this pandemic are yet to be determined. Alarmingly, not much is known about whether SARS-CoV-2 infection and Long COVID-19 might contribute to the shift of homeostasis into oncopathology or chronic fibrosis of the FRS as uterine fibrosis (UF), and there is a lack of publications on this subject. Due to the longitudinal nature of epidemiological studies of such a nature, and given the novelty of the SARS-CoV-2 coronavirus, there are no data yet about the potential impact of COVID-19, Long COVID-19, or their long-term sequelae on the risk of oncopathology or chronic fibrosis of the FRS and their pathogenesis. It is important to understand that COVID-19 will lead to a shift in homeostasis not from the point of conditional absolute health, but from the point of the current background shift of various systems, tissues, and regulatory backgrounds. We reviewed the recent literature on this topic and discuss potential changes in the FRS upon coronavirus entry in combination with the sum of background-state factors of various components of the tissue microenvironment. 

## 2. Impact of COVID-19 on the Microbial Environment—Bacteriome and Viriome

The microbiome and viriome are subtle but important components of the tissue microenvironment that influence tissue homeostasis. Until recently, it was believed that “respiratory infections” very rarely cause complications and should not be taken into account when predicting possible recurrences of a persistent infection, such as a viral one.

At the same time, an increasing number of observations indicates a significant impact of transferred COVID-19 on the state of the microbial environment. It has been shown that SARS-CoV-2 infection can lead to reactivation of oncogenic viruses in tissues. For example, a severe form of COVID-19 causes reactivation of cytomegalovirus (CMV) and the herpes simplex virus (HSV) [6] (Table 1). 

At the same time, in an in vitro study on cell lines (human umbilical vein endothelial cells (HUVEnCs), human colorectal adenocarcinoma cells (Caco-2), and retinal pigment epithelial cells (RPE-1s)) using blood serum from patients with a history of previous human cytomegalovirus (HCMV) infection significantly increases the possibility of SARS-CoV-2 entry into cells by activation of ACE2, the SARS-CoV-2 cell entry receptor [10].

COVID-19 also induces some human endogenous retroviral elements (HERVs), replication- and retrotransposition-defective sequences of ancient viral origin integrated into the human genome millions of years ago. It has been described that SARS-CoV-2 induces expression of the envelope protein of the human endogenous retrovirus type W in blood lymphocytes and tissues of patients with COVID-19 [11]. HERVs are known to promote development of cancer features, including genome instability [12]. Increases in the levels of some HERVs is a molecular characteristic of endometrial carcinoma (EC); for example, the HERV-W envelope gene syncytin-1 (which, in particular, is required for human placental morphogenesis due to its regulatory role in formation of syncytia) is significantly increased in EC [13]. Notably, cell fusion involving cancer cells and mediated by HERVs plays an important role in cancer initiation and progression [14]. Although the causal roles of the herpes simplex virus (HSV) and cytomegalovirus (CMV) in EC are unclear, it has been suggested that they may be involved in the pathogenesis of EC [15,16]. A role for HERVs in pulmonary fibrosis has also been hypothesized [17]. However, given the novelty of SARS-CoV-2 infection, nothing is known about the interaction of EC, UF, and COVID-19 in such a context. Thus, it may be of interest to evaluate whether COVID-19 affects HERVs and oncogenic viruses in EC and UF and whether they can be used as prognostic or therapeutic biomarkers in the clinic.

COVID-19 may also lead to alteration of the FRS microbiome [7]. For example, it changes the composition of the vaginal microbiome, causing dysbacteriosis with significant reductions in some taxa and increases in others: in particular, an increase in bacteria of the Bacteroidota type (*p* = 0.018) and a decrease in the genus Lactobacillus (*p* = 0.007). When subgroups were analyzed, the number of Ureaplasma spp. was higher in women with moderate/severe disease than in women with asymptomatic/mild disease [8]. Although the uterus is a low-microbial site, it is not free from bacteria [18]. The endometrial microbiome has been fully characterized, and pathological conditions such as EC have been demonstrated to be associated with significant changes in microbiota [19].

It is to be expected that changes in the normal microbiome lead to chronic inflammation, which can also cause fibrotic changes as well as open up new relationships with development of FRS pathologies [20].

## 3. The Impact of COVID on Comorbidities and the Background State of the Body

As is known, COVID-19 is a systemic disease, or, according to [21], “COVID-19 is a multi-organ aggressor”. Case reports have shown multiple systemic effects of COVID-19 infection, including acute respiratory distress syndrome, fibrosis, colitis, thyroiditis, demyelinating syndromes, and mania, indicating that COVID-19 can affect most human body systems [22,23]. The most susceptible to the severe course of the disease are the elderly and people with concomitant diseases. It is known that SARS-CoV-2 affects men more strongly, which is associated with the level of hormones that affect expression of the ACE2 receptor [24]. It has been demonstrated that ACE2 levels are reduced in patients with diabetes mellitus, and at the same time, a history of diabetes is a risk factor for COVID-19 [25].

Some of these risk factors, such as older age, obesity, and sex hormone levels, are among the risk factors not only for COVID-19 but also for EC and UF [26,27,28]. However, there is a surprising reduction in the risk of UF among people with diabetes, whereas having a history of diabetes is a risk factor for COVID-19 and EC [29]. Development of diabetes mellitus, maternal adiposity, and insulin-dependent gestational diabetes are associated with COVID-19 in pregnancy [30]. A case of hypothalamic amenorrhea after SARS-CoV-2 infection in a 36-year-old healthy woman has been described [31]. As in the olfactory epithelium, high levels of ACE2 and TMPRSS2 are found in the ovaries and endometrium [32] and the luminal and glandular epithelial cells of the FRS [33]. It is also known that ACE2 levels in the endometrium fluctuate depending on the phase of the menstrual cycle [34]. ACE2 is widely expressed in human myometria and uterine leiomyoma [35]. Another study showed that ACE2 and TMPRSS2 are also expressed in EC cells [36]. Finally, ACE2 levels have been shown to be elevated in EC compared to in adjacent noncancerous tissue [37]. This gives reason to pay close attention to the development of possible pathological processes in the female system when it is infected with the SARS-CoV-2 virus.

A large body of research addresses the practical issue of the persistence of the SARS-CoV-2 virus in the FRS. Prospective studies indicate both the presence [38] and absence of the virus in the lower genital tract [39]. Such interest is primarily due to assessment of the impact of the virus on the course of pregnancy and the safety of IVF procedures. A multicenter study of 906 couples undergoing IVF showed that resulting COVID-19 infection prior to egg retrieval had no clear negative impact on egg and embryo outcomes, including the number of eggs retrieved, the rate of egg maturation, the normal fertilization rate, or the number of good-quality embryos. However, the results of linear regression in that study showed that the utilization rate of eggs retrieved after 7 days after infection was higher [40]. In addition, the subsequent identification of alternative pathways for the virus to enter, as well as symptoms associated with development of inflammation, cytokine storms, and microvascular damage, means that parallel pathogenetic processes occur in the body during infection with COVID-19. Thus, endothelial dysfunction of the vasculature due to COVID-19 is observed throughout the body [41] and can subsequently affect multiple organs, including FRS organs, regardless of the levels of ACE2 in the cells that make up the affected organ. In addition, in studying the placenta, in some cases (especially after preterm birth), complications were found in the form of diffuse and localized SARS-CoV-2 placentitis (infection of the placenta) [42]. All placentas showing the three classic features, diffuse villous agglutination, placental syncytiotrophoblast (ScT) necrosis, and histiocyte-dominated inflammatory infiltrate with varying amounts of perivillous fibrin, were found to be positive for SARS-CoV-2 with immunohistochemistry (IHC). Overall, we agree with the view presented by Saadedin et al. that although ACE2 is expressed in FRS tissues, its role is not predominant in the adverse effects of COVID-19 on the FRS [22]. However, given the complex system of tissue regulation of the FRS and the potential for asymptomatic and incidental detection of endometrial cancer, such as in spontaneous abortion [43], more information needs to be collected about negative effects as well as the positive ones. As an example, a case of spontaneous tumor regression after vaccination against COVID-19 was described [44].

## 4. Main Events in the Systemic Circulation under the Influence of COVID-19

One of the mechanisms underlying the systemic impact of COVID-19 is a change in the homeostasis of the immune system, including changes in the levels of cytokines, growth factors, metabolites, and lipoproteome [45]. In addition to the above, in the systemic circulation, the presence of a soluble form of ACE2 has been determined [46]; it is also capable of binding SARS-CoV-2 and somehow facilitating penetration of SARS-COV-2 into cells [47]. Its levels increase significantly at the onset of COVID-19 [48], and it has been suggested that the complex of soluble ACE2 with SARS-CoV-2 can enter cells through receptor-independent macropinocytosis [47]. In addition, the virus envelope proteins themselves are persistent, as are immune-influencing agents—the nucleocapsid protein (N-Ag) of the coronavirus in serum [49]. It has been shown that extracellular soluble S1 protein is a key viral component inducing pro-inflammatory responses in macrophages, independent of virus replication. Thus, virus- or soluble S1-activated macrophages may become sources of pro-inflammatory mediators contributing to hyperinflammation in COVID-19 patients [50].

For example, the potential amyloidogenicity of the SARS-CoV-2 S protein, which causes formation of amyloid fibrils in vitro, as a result of viremia, can lead to formation of fibrin-amyloid microcells in the bloodstreams of individuals affected by COVID-19 and can lead to blockage of capillaries in various tissues [51].

Using mass cytometry and multiplex immunoassays, scientists have determined more than 170 immunological parameters, including 49 markers of inflammation—cytokines, cytokine receptors, chemokines, and growth factors. HGF and CXCL13 demonstrated the highest sensitivity and specificity, respectively. Elevated levels of HGF and CXCL13 have been associated with risk of hospitalization and death. At the same time, the combination of molecules has a greater predictive power than each molecule separately [52]. New data on identification of metabolites and cytokines predictive of outcomes for patients with severe SARS-CoV-2 infection have shown similarities with cancer. Multivariate analysis has shown that low levels of HGF, lactate, and phenylalanine are correlated with good outcomes [53].

The onset of COVID-19 triggers the host’s innate and adaptive immune systems, resulting in secretion of a wide range of proinflammatory cytokines. Accumulating evidence has indicated that infection with SARS-CoV-2 leads to increased levels of proinflammatory cytokines such as IL-6, IL-1β, TNF-α, and IFN-γ, known as molecules “at the intersection of COVID-19 and cancer” [54] and also known to be involved in formation of fibrous tissue, thus creating an oncogenic and profibrotic inflammatory microenvironment.

Plasma levels of IL-1β (presumably among others) have been shown to be elevated, at least temporarily, not only during the acute phase of SARS-CoV-2 infection but even in individuals who have recovered from COVID-19 [55]. Inflammation plays a significant role in the pathogeneses of many types of cancer, including EC. For example, it has been shown that IL-6 and TNF-a can contribute to development of EC [56,57] and that their levels are elevated in EC tissue [58].

IL-6 also affects the levels of PD-L1 expressed by EC cells, resulting in escape from immune surveillance [59,60]. PD-L1 expression can be induced by IFNy [61], one of the cytokines elevated during the acute phase of COVID-19, and the PD-1/PD-L1 axis has been shown to be modulated by COVID-19. Regulatory microRNA miR-15-5p-targeting PD1 was found in the CD8+ T cells of patients who recovered from COVID-19, indicating changes in the PD-1/PD-L1 immune checkpoint axis [62]. However, the question of how COVID-19 modulates PD-1/PD-L1 in EC is still open.

Notably, immunological dysfunction caused by COVID-19 is a long-term phenomenon, persisting up to 8 months after infection [63] and at least partially caused by long-term epigenomic changes, such as changes in the three-dimensional organization of the genome [64].

## 5. Events during Penetration of SARS-CoV-2 at the Tissue Level

When a virus enters, it is important to evaluate the mutual processes occurring at the tissue level, since they can be the material substrate of clinical manifestations that may be observed in the future. It is important to understand not only how the virus enters cells, but also the molecular shifts in tissue regulatory molecules secreted by the cells, as well as the resulting rearrangements of the extracellular matrix (ECM).

ACE2 is considered to be the central cellular receptor used by SARS-CoV-2. ACE2 is a host cell glycoprotein receptor that mediates entry of SARS-CoV-2 into the cell by interacting with the receptor-binding domain (RBD) of the surface spike glycoprotein (S-protein) of the coronavirus. The main function of ACE2 in the body is its role in the renin–angiotensin–aldosterone system (RAS), the most important regulator of cardiovascular homeostasis [65]. There are several isoforms of ACE2, including a transmembrane ACE2 capable of S-protein binding and a secreted form of ACE2 that is incapable of such activity [66]. SARS-CoV-2 infection has been shown to affect ACE2 levels, although not in a unidirectional manner. First, they may be elevated in response to inflammatory signaling [67]. Second, the levels of ACE2 receptors on the cell membrane may decrease due to coronavirus infection [68], possibly due to its cellular internalization in complex with the virus.

Another important molecular player in the SARS-CoV-2 cellular internalization process is the TMPRSS2 proteinase. The SARS-CoV2 S protein mentioned above contains two noncovalently linked and functionally distinct subunits: the S1 subunit, which binds to ACE2, and the S2 subunit, which is involved in fusion of the virus with the cell membrane [69]. Priming of the SARS-CoV-2 S protein TMPRSS2 on the cell membrane activates it for host-cell-membrane fusion and is vital for virus entry into target cells [70]. TMPRSS2 is considered to be the predominant proteinase that cleaves the S2 subunit of the SARS-CoV-2 S protein, which is necessary for its entry into the cell [36]. Although ACE2 and TMPRSS2 are the key molecules used by SARS-CoV-2 to enter the cell, there are also TMPRSS2-independent entry mechanisms [71,72]: for example, cathepsin B/L, which carries out its cleavage in the endosomal compartment [73]. In addition, other proteases besides TMPRSS2, such as cathepsin B/L and others, are involved in infection of the SARS-CoV-2 target cell [74], and the list of such molecules is expected to grow continuously as we continue to study the biology of SARS-CoV-2. Moreover, ACE2-independent mechanisms of SARS-CoV-2 entry into cells have also been described [75,76,77].

For example, it is hypothesized that SARS-CoV-2 may use an extracellular matrix metalloproteinase inducer (EMMPRIN, also known as CD147) as a cell entry receptor [76]. EMMPRIN is expressed by many cell types and is known to be one of the key regulators of cancer-cell invasion and metastasis and an emerging regulator of cancer signs [78].

Interestingly, its level is elevated in patients with endometriosis [79]. It also plays a role in endometrial carcinoma (EC) [9] and is known to be involved in fibrosis, including uterine fibroids [80].

Another biomolecule potentially involved in ACE2-independent entry of SARS-CoV-2 into host cells is the receptor tyrosine kinase AXL [76], which is downregulated in uterine myoma compared to normal myometria [81] but is highly expressed in the endometrium and is involved in the pathogenesis of EC [82].

Notably, both AXL and EMMPRIN regulate extracellular-matrix (ECM) remodeling, a process closely associated with the pathogeneses of EC and UF and one of the known consequences of COVID-19. Several groups have recently conducted in-depth reviews and reported that the ability to fuse host cells is a hallmark of SARS-CoV-2 [83,84,85].

Thus, it can be hypothesized that there may also be other hypothetical and as-yet untested scenarios for SARS-CoV-2 entry into the cell, such as entry through cell fusion into a multinucleated syncytium, involving fusion of infected cells expressing ACE2 and uninfected cells without such a receptor or at a low level, followed by budding of infected cells from the syncytium.

Notably, it has been shown that S-protein-induced intercellular fusion can be inhibited by gefitinib, one of the epidermal growth factor (EGFR) inhibitors. At the same time, the EGFR is known to be one of the main regulators of EC cell growth [86], which may provide a biological rationale for targeting the EGFR in EC with high EGFR levels and comorbid COVID-19. This may be of clinical importance in determining AXL and EMMPRIN levels in normal, precancerous, and malignant endometria, as well as in normal and fibrous uterine tissues.

The exact molecular mechanisms behind the etiology and pathogenesis of UF remain somewhat elusive, but the condition has known risk factors that shed some light on its possible causes. Among the known risk factors for UF that are also associated with COVID-19 are changes in the function of the RAS (renin–angiotensin system) [87], and a contribution of the RAS to the pathogenesis of EC has also been proposed [37]. In addition to being a receptor for SARS-CoV-2, ACE2 is also involved in regulation of homeostasis through RAS modulation. Thus, any changes in the levels of ACE2, in the expression spectrum of its isoforms, in their distribution on the cell surface, in the secretion of ACE2, and so on will subsequently affect its “canonical” role as a regulator of the RAS.

## 6. Impact of COVID-19 on the Extracellular Matrix

SARS-CoV-2 induces several growth factors associated with tissue fibrosis. In patients with moderate severity of COVID-19, TGF-α, FGF-basic, and EGF levels were shown to be significantly elevated but decreased with progression of severity [88]. An increase in the level of TGF-β under the influence of SARS-CoV-2, which led to remodeling of the vascular extracellular matrix, was also shown [89]. In studying the placenta, in a number of cases (especially after preterm birth), complications in the form of diffuse and localized SARS-CoV-2 placentitis (infection of the placenta) were found in patients with COVID-19. All placentas showing the three classic features of placentitis, diffuse villous agglutination, placental syncytiotrophoblast (ScT) necrosis, and histiocyte-dominated inflammatory infiltration with varying amounts of perivillous fibrin, were found to be positive for SARS-CoV-2 with immunohistochemistry (IHC) [42]. The outcomes of such inflammation are also tissue fibrosis and remodeling of the extracellular matrix.

COVID-19, through metabolic changes, also affects the so-called advanced-glycation end products/receptor for the advanced-glycation end-products (AGE/RAGE) axis in the host [90]. RAGE is a well-known driver of inflammation and contributes to the “oncogenic” niche. The ECM is the cornerstone of the tissue microenvironment and one of the main regulators of cell cross-talk in response to microenvironmental stimuli. The unique characteristics of the ECM are genuine characteristics of EC and UF. For example, some components of the ECM, such as aggrecan, nidogen, α1-chain collagen type VIII, and α2-chain collagen type XI, are elevated in stage III EC. However, the molecular, cellular, and mechanical features of the ECM at different stages of disease initiation and progression, from a normal endometrium to precancerous and malignant EC tissue, remain elusive [91]. Generally, any fibrosis is attributed to excessive deposition of the ECM [92] and changes in its composition and mechanical characteristics. In the case of UF, the composition of the ECM changes, and the contents of collagen 1A1, fibronectin, and versican in the ECM increase [93]. The aberrant ECM of UV contributes to its pathogenesis, at least as it has been demonstrated in relation to the role of directed ECM mechanotransduction and the central part of ECM rigidity in the pathogenesis of UV [94]. Pulmonary fibrosis caused by SARS-CoV-2 infection is very well-understood and its mechanisms can be extrapolated to UF to some extent. However, the tissue microenvironment of UF is unique in terms of its ECM, immune-cell infiltration, the hormonal environment and its changes, and so on. The literature has demonstrated that COVID-19 significantly affects ECM remodeling in lung tissue [95,96], but long-term effects on the FRS, including in EC and UF, are unknown and should be investigated.

## 7. Reversible Cellular Transitions—Polarization of Macrophages, Influence on EMT and EnMT

The cells that make up the tissue niche can go through various types of reversible cellular shifts; they are part of both normal and pathological processes. These may include reversible changes in polarization of macrophages and epithelial–mesenchymal transitions and endothelial–mesenchymal transitions of cells. SARS-CoV-2 may indirectly influence the above processes through inflammation and hypoxia of SARS-CoV-2 [97].

Macrophages, the mononuclear cells of the innate immune system, may facilitate or counteract inflammation processes, depending on their phenotype and upstream signaling. Once recruited into the microenvironment, they can acquire different phenotypes. Macrophages can be subdivided by phenotype into nonactivated macrophages (M0), classically polarized macrophages (M1), and, alternatively, polarized macrophages (M2), which can be further subdivided into M2a, M2b, and M2c. The M1 type is proinflammatory while the M2 type is an anti-inflammatory phenotype. It is hypothesized that these phenotypes exhibit a certain degree of plasticity, M1 can acquire the M2 phenotype and vice versa, and cells can fluctuate between these states. Simply put, M2 macrophages are primarily involved in angiogenesis, tissue repair, and remodeling, while M1 macrophages mediate resistance to various pathogens. Historically and in accordance with modern concepts, M2 macrophages are considered protumor cells [98]. In the tumor microenvironment (TME), tumor-associated macrophages (TAMs) promote tumor progression and metastasis and modulate immunosuppression and tumor-cell evasion from immunological surveillance. In EC, TAMs penetrate tumor tissue. Tumor tissue of both EC types, I and II, has been shown to have an increased number of macrophages compared to the density of macrophages in benign endometria [99]. The interaction of EC cells and associated TAMs is an important part of the pathogenesis of EC. Despite the fact that uterine endometrial macrophages are predominantly of the M2 type and the M2b subtype [100], under conditions of hypoxia, EC cells can polarize monocytes to M2-like macrophages [62], which can further shift polarization of macrophages within EC toward the phenotype, promoting tumor development, and this needs to be investigated. Overall, COVID-19 and prolonged COVID-19 may promote the formation of a proinflammatory niche, in the microenvironment of uterine tissue, that is profibrotic and oncogenic. At the same time, it can change the phenotypes of TAMs, affecting the growth and progression of tumor tissue.

The endometrium is a tissue where the epithelial–mesenchymal transition (EMT), the process by which epithelial cells differentiate into mesenchymal (fibroblast-like) cells, is part of the tissue’s normal physiological function as it undergoes repeated proliferation and shedding during the menstrual cycle. Significant changes in this environment include fluctuations in oxygen partial pressure, exposure to a high-cytokine environment associated with intrauterine infection, and inflammation. In a study on endometrial cell lines, it was shown that proinflammatory cytokines caused a significant increase in the level of HIF-1α mRNA and increased hypoxia-induced accumulation of the HIF-1α protein. The combined effect of inflammatory cytokines and hypoxia increased the expression of EMT-inducing factors and enhanced cell migration [101]. Moreover, the SARS-CoV-2 spike protein itself has been found to increase the metastatic potential of breast cancer through activation of Snail, one of the modulators of EMT [102], and leads to an increase in the levels of EMT genes in breast-cancer cells, causing malignant transformation [103]. The blood serums of patients who recovered from COVID-19 had the same effect on cells [104].

Endothelial–mesenchymal transition (EndMT) occurs similarly to EMT: when vascular cells and endothelial cells enter the mesenchymal state. This requires damage to the vascular endothelium, chronic inflammation, and the presence of proinflammatory cytokines, which occur with COVID-19 and lead to endothelial dysfunction [105].

Using single-cell RNA sequencing, we profiled cell transcriptomes from the lungs of healthy individuals, patients with COVID-19, and patients with idiopathic pulmonary fibrosis and identified distinct subpopulations of endothelial cells (EnCs) and epithelial cells as major cellular sources of MT accumulation during COVID-19 [106].

## 8. Impact of COVID-19 on the Genome and Epigenome

A sufficient amount of data has been accumulated about COVID-19 through the serum of patients [104], showing that increased levels of growth factors affect gene expression [86]. There are differences in endometrial gene expression in women with coronavirus disease 2019 [107]. The SARS-CoV-2 spike glycoprotein shares 41 minimal immune determinants, i.e., pentapeptides, with 27 human proteins that are associated with oogenesis, uterine receptivity, decidualization, and placentation. All but four of the common pentapeptides we have identified are also present in epitopes derived from the SARS-CoV-2 glycoprotein, which have been experimentally confirmed to be immunoreactive [108].

Noncoding RNA (ncRNA) plays a fundamental role in various biological processes associated with the pathogenesis of endometrial cancer, and many varieties also have a prognostic function, which is of great importance for determining therapeutic pathways and patient follow-up. Personalized medicine focuses on constantly updating risk factors that are identified in the early stages of endometrial cancer in order to tailor treatment to patients [109,110].

Regulatory noncoding RNA (NCRNA), such as miRNA (miRNA) and long noncoding RNA (lncRNA), plays a vital role in EC oncogenesis and UF pathogenesis [94,111] and can therefore be used as EC/UF biomarkers or therapeutic targets. Many varieties may be affected by COVID-19. It has been reported that some microRNA can modulate TGF-β pathways in UF and myometrial cells [112], so it would be interesting to evaluate its levels during and after COVID-19. Interestingly, one recent paper identified several miRNAs differentially expressed in individuals affected by COVID-19, and KEGG pathway-enrichment analysis identified the endometrial cancer pathway (hsa05213) as one of the common pathways for miRNA to up- or downregulate COVID-19 [113].

SARS-CoV-2 has been shown to cause DNA damage and general genome instability [114,115], well-known hallmarks of cancer and driving forces behind tumorigenesis. COVID-19 may be a risk factor. A recent publication demonstrated that an increased risk of COVID-19 is associated with an increased risk of EC [116].

Studies of genetic susceptibility to UF suggest that genes involved in regulation of genome stability may somehow contribute to the pathology of UF [117], while COVID-19, as discussed above, leads to genome instability. Interestingly, individuals with UF often carry mutations in MED12 gene-encoding subunit 12 of the mediator complex (MED12) [118]; such mutations have been shown to be present in UF stem cells but not in stem cells of adjacent normal myometria, and stem cells of UF with mutations in MED12 have shown increased DNA damage [119]. Other studies have shown that MED12 is simultaneously associated with DNA damage repair (DDR) and is found in the SARS-CoV-2 protein interactome [120]. Thus, it is possible that COVID-19 will exacerbate UF.

It has recently been demonstrated that SARS-CoV-2 disrupts chromatin regulation in infected cells [121]. At the same time, UF is characterized by epigenetic changes, such as insufficient deposition of histone H2A.Z, which leads to disruption of the differentiation program in cells [122]. H2A.Z is also critical for repair of double-stranded DNA damage [123]. In EC, the chromatin-remodeling and DDR genes are also frequently mutated, which is described in detail elsewhere [124] and is therefore beyond the scope of this review. Overall, it can be hypothesized that SARS-CoV-2, through its interference with the chromatin structure and DDR molecular machinery, may “aggravate damage” in DDR-compromised cells.

## 9. Conclusions and Future Directions

**Figure 1:**
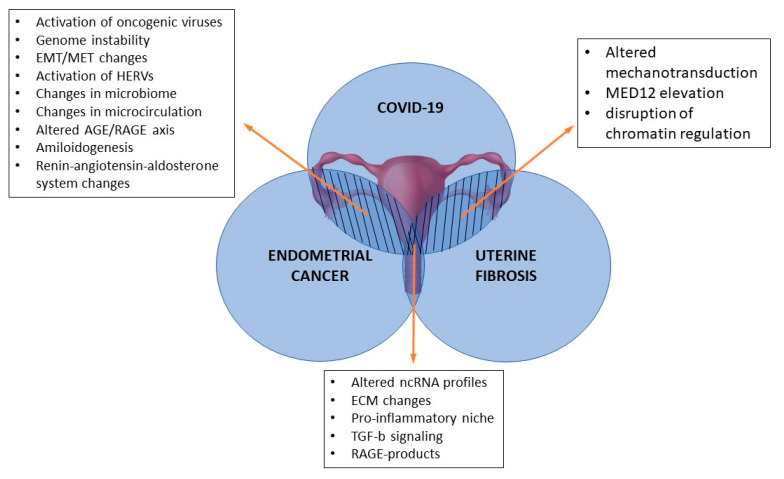
Possible molecular mechanisms inducing shift of homeostasis into endometrial cancer or uterine fibrosis as a result of COVID-19.

Evidence regarding impacts of COVID-19 on multiple other diseases continues to mount, but there are significant gaps in the current knowledge on this subject, given the novelty of SARS-CoV-2. In this concise review, we have summarized the latest publications’ statements about the potential roles of COVID-19 in EC and UF pathogenesis (Figure 1).

Specific significant markers can be considered in predicting certain pathologies in patients who have undergone COVID-19 (Table 2). This comparison is helpful for better interpretation of changes in the expression levels of specific markers, the dynamics of which may differ as well as coincide for the diseases as mentioned above, masking one as the other.

We hypothesized that EC and UF might be COVID-19 sequelae, speculated about possible mechanisms underlying such potential phenomena, and proposed several experiments that may test this hypothesis. Further studies of the role of COVID-19 as a contributor to and regulator of the molecular processes underlying EC and UF will likely provide novel insights into their aetiologies and pathophysiologies.

## Figures and Tables

**Table 1:** Microbiome changes by COVID-19.

Microbiome	Changes by COVID-19
Cytomegalovirus (CMV) and Herpes simplex virus (HSV)	Are reactivated [6]
Firmicutes	Significant decrease in microbiome [7]
Bacteroidota	Increase [7,8,9],
Lactobacillus	Decrease [7], decrease after menopause [8]
*L. crispatus*, *L. iners*, *L. gasseri*, and *L. jensenii*	Relative abundance was lower [7]
Ureaplasma	The amount was higher in women with moderate/severe than with asymptomatic/mild disease [7]; increase [8]

**Table 2:** Markers affecting/experiencing change as a result of COVID-19 disease and fibrosis/cancer pathogenesis.

Marker	COVID-19	Fibrosis	Cancer
ACE2	Level of soluble ACE2-creating complex with COVID-19 increases at the onset of the disease [47,48]		Levels of ACE2 are increased in EC compared to adjacent noncancerous tissue [37]
	Can be upregulated in response to inflammatory signaling [67]		
	Levels of the ACE2 receptor on the cellular membrane may decrease due to coronavirus infection [68]		
	Levels of ACE2 are lowered in diabetic patients, and at the same time, having diabetes in anamnesis is a risk factor for COVID-19 [25]		
TMPRSS2	Priming of SARS-CoV-2 S-protein by TMPRSS2 at the cellular membrane activates it for host-cell-membrane fusion [70]	High in periconceptional human endometrium [33]	Expression level is high in EC cells [36]
Cathepsin B/L	Are involved in SARS-CoV-2 infection of the target cell [74]	-	-
EMMPRIN (CD147)	A cell entry receptor [8]	Level is elevated in patients with endometriosis [79] involved in fibrosis including uterine fibroids [80]	Significant in EC [9]
Receptor tyrosine kinase (AXL)	Potentially involved in ACE2-independent SARS-CoV-2 entry into the host cell [76]	Is downregulated in uterine fibroids compared to normal myometria [81]	Highly expressed in endometria and involved in EC pathogenesis [82]
Cytomegalovirus (CMV) and herpes simplex virus (HSV)	Are reactivated [6]		May be involved in EC pathogenesis [15,16]
Some human endogenous retroviral elements (HERVs)	Induced [12]	The role of HERVs in pulmonary fibrosis has also been suggested [17]	Contribute to development of the hallmarks of cancer [12]
			Level of Syncytin-1 is significantly increased in EC [13]
			Play important roles in cancer initiation and progression [14]
IL-6, IL-1β, TNF-α, IFN-γ	COVID-19 causes elevated levels of these cytokines [8,53]		IL-6 and TNF-a can promote EC [55,56]
	Plasma levels of IL-1β are increased during the acute phase of SARS-CoV-2 infection and in individuals who have recovered from COVID-19 [54]		IL-6 and TNF-a levels are elevated in EC tissue [57]
			IL-6 also affects levels of PD-L1 expressed by EC cells [58]
PD-1, PD-L1	In CD8+ T cells of patients recovered from COVID-19, PD1-targeting regulatory microRNA miR-15-5p was detected, suggesting changes in the PD-1/PD-L1 immune checkpoint axis [54]		In EC PD-L1, tumor-cell expression profiles are different in molecular and histologic subtypes [60]
			Can be induced by IFNγ [61]
Macrophages	Hypoxia is one of the consequences of being infected, and it may shift polarization of macrophages within EC further toward the tumor-promoting phenotype [97]		Tumor tissue of EC has elevated numbers of macrophages compared to the density of macrophages in the benign endometrium [99]
			Under hypoxic conditions, EC cells can polarize monocytes to M2-like macrophages [62,100]
ECM components	Have significant impact on ECM remodeling in lung tissue [95,96]	Collagen 1A1, fibronectin, and versican are elevated [93]	Aggrecan, nidogen, collagen type VIII chain α1, and collagen type XI chain α2 are elevated in stage III EC [91]
		Roles in ECM-directed mechanotransduction and the central role of ECM stiffness in the uterine-fibrosis (UF) pathogenesis [94]	
RAGE	SARS-CoV-2 can lead to hyperactivity of RAGE in several cell types [90]		Are elevated in EC and predominantly low in healthy tissue [90]
TGF-β	Elevated [89]		
Genetic factors	SARS-CoV-2 causes DNA damage and overall genome instability [114,115]		
	Some miRNA affected by COVID-19 can modulate TGF-β pathways in UF and myometrial cells [103,112]		
Mediator complex subunit 12 (MED12)	MED12 is both linked to DNA damage repair (DDR) and found in the SARS-CoV-2 protein interactome [120]	UF often carries mutations in the MED12 gene [118]	
		UF stem cells with mutations in MED12 demonstrate increased DNA damage [119]	
Chromatin	SARS-CoV-2 disrupts chromatin regulation in infected cells [121]	Is characterized by deficient deposition of H2A.Z histone [122]	Chromatin remodeling and DDR genes are also frequently mutated [124]
Noncoding RNA		Vital roles in UF pathogenesis [94,111]	Vital roles in EC tumorigenesis [94,111]

## Data Availability

Not applicable.
